# De-escalation of axillary surgery in breast cancer patients treated in the neoadjuvant setting: a Dutch population-based study

**DOI:** 10.1007/s10549-020-05589-3

**Published:** 2020-03-16

**Authors:** J. M. Simons, L. B. Koppert, E. J. T. Luiten, C. C. van der Pol, S. Samiei, J. H. W. de Wilt, S. Siesling, M. L. Smidt

**Affiliations:** 1 Department of Surgical Oncology, Erasmus MC Cancer Institute, Rotterdam, The Netherlands; 2 Department of Surgical Oncology, University Medical Center Utrecht, Utrecht, The Netherlands; 3 GROW – School for Oncology and Developmental Biology, Maastricht University Medical Center, Maastricht, The Netherlands; 4 Department of Surgical Oncology, Amphia Hospital, Breda, The Netherlands; 5 Department of Surgical Oncology, Alrijne Hospital, Leiderdorp, The Netherlands; 6 Department of Surgical Oncology, Maastricht University Medical Center+, Maastricht, The Netherlands; 7 Department of Surgery, Radboud University Medical Centre, Nijmegen, The Netherlands; 8 Department of Research, Netherlands Comprehensive Cancer Organisation, Utrecht, The Netherlands; 9 Department of Health Technology and Services Research, Technical Medical Centre, University of Twente, Enschede, The Netherlands; 10 Department of Surgery, University Medical Center Utrecht, P.O. Box 85500, 3508 GA Utrecht, The Netherlands

**Keywords:** Breast cancer, Node positive, Sentinel lymph node biopsy, Axillary lymph node dissection, Axillary staging, Marked node

## Abstract

**Purpose:**

An overall trend is observed towards de-escalation of axillary surgery in patients with breast cancer. The objective of this study was to evaluate this trend in patients treated with neoadjuvant systemic therapy (NST).

**Methods:**

Patients with cT1-4N0-3 breast cancer treated with NST (2006–2016) were selected from the Netherlands Cancer Registry. Patients were classified by clinical node status (cN) and type of axillary surgery. Uni- and multivariable logistic regression analyses were performed to determine the clinicopathological factors associated with performing ALND in cN+ patients.

**Results:**

A total of 12,461 patients treated with NST were identified [5830 cN0 patients (46.8%), 6631 cN+ patients (53.2%)]. In cN0 patients, an overall increase in sentinel lymph node biopsy (SLNB) only (not followed by ALND) was seen from 11% in 2006 to 94% in 2016 (*p* < 0.001). SLNB performed post-NST increased from 33 to 62% (*p* < 0.001). In cN+ patients, an overall decrease in ALND was seen from 99% in 2006 to 53% in 2016 (*p* < 0.001). Age (OR 1.01, CI 1.00–1.02), year of diagnosis (OR 0.47, CI 0.44–0.50), HER2-positive disease (OR 0.62, CI 0.52–0.75), clinical tumor stage (T2 vs. T1 OR 1.32, CI 1.06–1.65, T3 vs. T1 OR 2.04, CI 1.58–2.63, T4 vs. T1 OR 6.37, CI 4.26–9.50), and clinical nodal stage (N3 vs. N1 OR 1.65, CI 1.28–2.12) were correlated with performing ALND in cN+ patients.

**Conclusions:**

ALND decreased substantially over the past decade in patients treated with NST. Assessment of long-term prognosis of patients in whom ALND is omitted after NST is urgently needed.

## Introduction

In breast cancer, systemic therapy is increasingly administered in the neoadjuvant setting [i.e., neoadjuvant systemic therapy (NST)] [1, 2]. One of the advantages of NST is the possibility of downsizing or even downstaging disease, which can occur in breast and/or axilla. In the best-case scenario, patients achieve a pathologic complete response (pCR), meaning that there is no histologic evidence of residual tumor. Downstaging is not only associated with a favorable prognosis but it also enables surgeons to opt for less extensive surgery after NST.

In clinically node-negative (cN0) patients, sentinel lymph node biopsy (SLNB) is widely accepted as primary regional staging procedure. In the case of a positive SLNB with limited tumor burden, it is safe to omit completion axillary lymph node dissection (ALND) in patients treated with lumpectomy in terms of disease-free and overall survival [3–7]. In the AMAROS trial, cT1-2N0 patients with a positive SLNB and treated with lumpectomy or mastectomy were randomly assigned to completion ALND or axillary radiotherapy [8]. The 5-year axillary recurrence rate was comparable in both groups, but measurable lymphedema occurred significantly less frequently in the axillary radiotherapy arm. The results of these trials resulted in decreasing use of ALND, as was proven by several cohort studies over the past years [9–12]. In these studies, patients treated with NST were not included and it is yet unknown if this decrease in axillary surgery also affects cN0 patients who were treated with NST.

In clinically node-positive (cN+) patients, axillary staging is an area of controversy. Traditionally, ALND was performed in all patients. However, at least 1 out of 3 cN+ patients treated with NST converts to a pathological node-negative axilla [13]. Since cN+ patients with an axillary pCR are not expected to benefit from ALND, different less invasive methods have been proposed to replace ALND, such as SLNB, MARI (marking the axillary positive lymph node with an iodine seed), and Targeted Axillary Dissection (i.e., a combination of the SLNB and a MARI-like procedure). However, since sufficient data are lacking on the outcome of cN+ patients in whom ALND is omitted after NST, broad implementation of less invasive staging procedures in clinical practice may be hampered.

With the increased use of NST in both cN0 and cN+ patients, and the de-escalation of axillary surgery in patients treated in the adjuvant setting, it is hypothesized that a trend of de-escalation will also be noticed in patients treated with NST. Therefore, the aim of this study was to evaluate the trends in de-escalation of axillary surgery in cT1-4N0-3 breast cancer patients treated with NST in the Netherlands.

## Methods

Data were collected from the Netherlands Cancer Registry (NCR) [14]. The NCR is hosted by the Netherlands Comprehensive Cancer Organisation (IKNL). Specially trained registration clerks gather data directly form the patient files in all hospitals in the Netherlands. Patients with cT1-4N0-3 breast cancer treated with NST, between 2006 and 2016, were included. Patients with occult breast cancer were also included. For each patient, the following variables were documented: hospital type (academic, teaching, community), age, morphological subtype, receptor status, TNM status prior to and after NST, NST regimens, type of breast, and axillary surgery, axillary pCR, and adjuvant treatment plans. Axillary pCR was defined as the absence of residual axillary disease in all examined lymph nodes independent of the type of axillary surgery. Isolated tumor cells were included in the definition of axillary pCR. Patients were excluded if no lymph nodes were identified during surgery or if the number of positive lymph nodes was unknown. Patients with unknown cN status, distant metastasis, or patients in whom surgery of the breast was not performed were also excluded.

### Patients

In the Netherlands, the axilla is generally assessed by means of ultrasound at the time of diagnosis. In the case of suspicious lymph nodes, either fine needle aspiration (FNAC) or core needle biopsy (CNB) is performed to assess the presence of metastasis. A cN0 status was defined as the absence of suspicious lymph nodes on axillary ultrasound or a negative FNAC/CNB (if performed). A cN+ status was defined as the presence of suspicious lymph nodes on axillary ultrasound in combination with pathologically confirmed metastasis by FNAC/CNB. Patients in whom SLNB was performed prior to NAC were always considered as initially cN0 patients.

For cN0 patients, the following subgroups regarding type of axillary surgery were documented: SLNB only (i.e., not followed by completion ALND), SLNB followed by completion ALND, and ALND (not preceded by SLNB). Patients in whom the timing of SLNB was unknown were excluded from analysis. For cN0 patients in whom SLNB was performed prior to NST, data on ypN status were only available when an ALND was performed.

For cN+ patients, the following subgroups were documented: SLNB and/or MARI only (i.e., not followed by completion ALND), SLNB and/or MARI followed by completion ALND, and ALND (not preceded by SLNB and/or MARI). MARI includes any procedure in which the pathologically confirmed positive lymph node was marked prior to NST and selectively removed after NST.

When referred to ypN status, this is always based on pathologic examination of lymph nodes and not on post-NST clinical examination of the axilla.

### Statistical analysis

Descriptive statistics were performed to evaluate the trends over time for omission of ALND in the overall population, in cN0 and cN+ patients. In addition, potential differences were explored in the axillary management associated with the type of hospital where patients were treated. Univariable and multivariable logistic regression analyses were performed to determine the clinicopathological factors associated with performing ALND in cN+ patients. Odds ratios (ORs) were presented with 95% confidence intervals (CIs). Two-sided *p* values of < 0.05 were considered statistically significant. Data analysis was performed using Stata/SE Statistical Software for Windows, version 14.2 (College Station, TX: StataCorp LP).

## Results

A total of 15,725 breast tumors treated with NST (10% of all breast tumors) were identified between January 2006 and December 2016 and registered in the NCR. Cases were excluded for the following reasons: distant metastasis (*n* = 683), treated with neoadjuvant radiotherapy (*n* = 21), unknown cN status (*n* = 165), unknown cT status (*n* = 136), unknown hospital type (*n* = 1), no breast surgery (*n* = 838), unknown type of axillary surgery (n = 674), unknown outcome of axillary surgery (*n* = 193), and unknown timing of SLNB (*n* = 553). Altogether, 12.461 breast tumors were included for the final analysis. See Table 1 for clinicopathologic characteristics. In 7106 of 12,461 (57%) cases, ALND was performed. From 2006 to 2016, an overall decrease in the rate of ALND was observed from 96 to 29% (*p* < 0.001).

**Table 1:** Clinicopathologic characteristics of all cT1-4N0-3 breast cancer patients treated with NST between 2006 and 2016 (*n* = 12.461)

Characteristics	*n* (%)
Year of diagnosis	
2006—2009	2286 (18.4)
2010—2013	4712 (37.8)
2014—2016	5463 (43.8)
Age in years, median (range)	50 (range 18–87)
Histologic subtype	
Ductal	9832 (79)
Lobular	1256 (10)
Adenocarcinoma NOS	550 (4.4)
Mixed ductal and lobular	307 (2.5)
Other	516 (4.1)
Receptor status	
HR−/HER2−	1751 (16.3)
HR−/HER2+	987 (9.2)
HR+/HER2+	1756 (16.4)
HR+/HER2−	6243 (58.2)
HR and/or HER2 status	1724 (13.8)
Unknown	
TNM status	
T0—1—2—3—4	14 (0.1)–1647 (13.2)–6595 (53)–2696 (21.6)–1509 (12.1)
N0—1—2—3	5830 (47)–5610 (45)–258 (2)–760 (8)
M0—X	12,351 (99)–110 (1)
Lumpectomy	5087 (41)
Mastectomy	7374 (59)
Neoadjuvant systemic regimen	
Chemotherapy only	9382 (75)
Chemotherapy with HER2 therapy	2732 (22)
Chemotherapy with endocrine therapy	286 (2)
Chemotherapy with HER2 and endocrine therapy	61 (1)

*NOS* not otherwise specified, *HR* hormone receptor, *NST* neoadjuvant systemic therapy

### cN0 patients

**Fig. 1:**
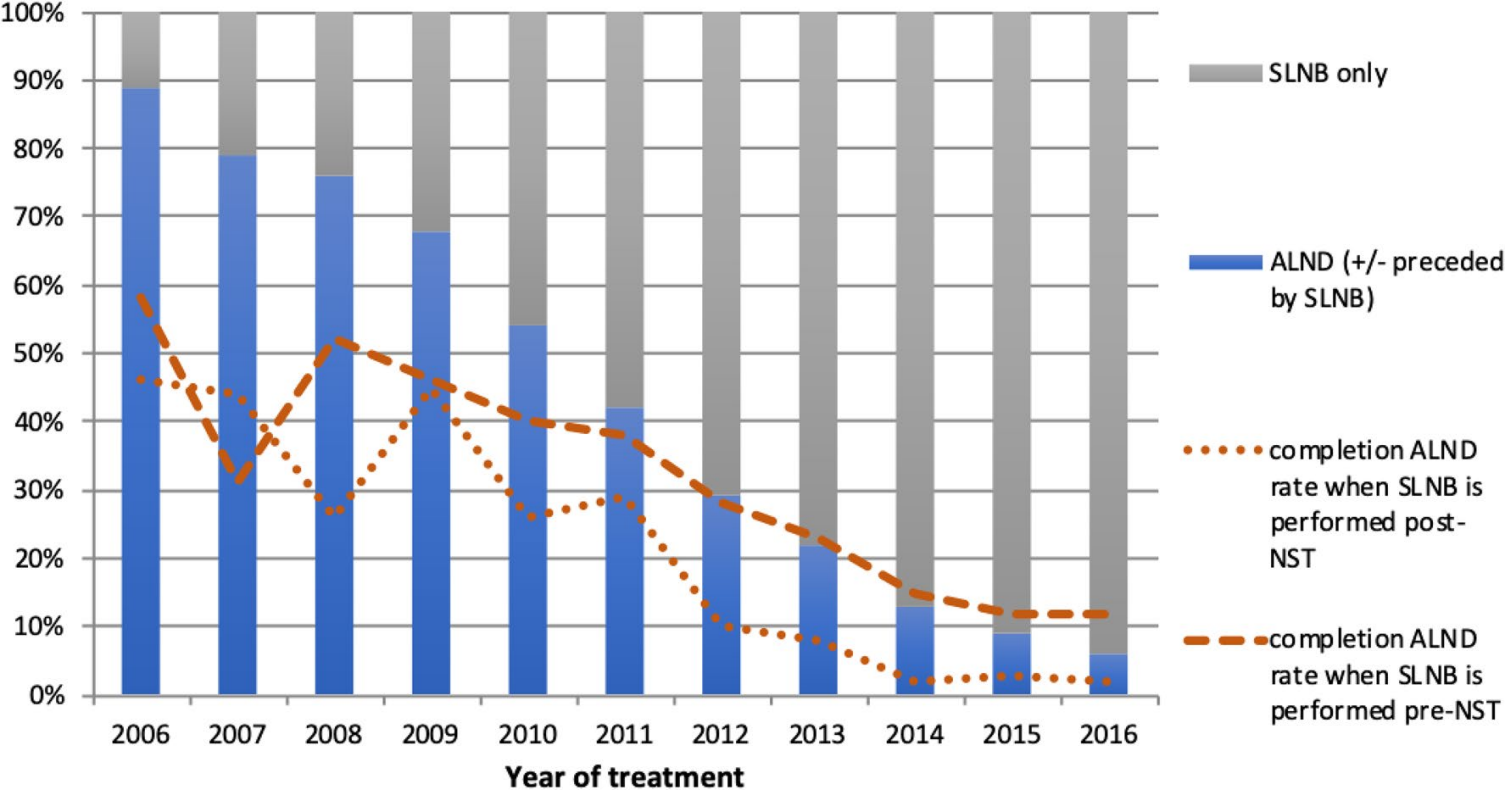
Course over time for axillary staging in cN0 patients treated with NST. *cALND* completion ALND

Nodal status was negative at the time of diagnosis (cN0) in 5830 cases (46.8%). The proportion of cN0 patients treated with NST increased from 35% in 2006 to 50% in 2016. In total, 4301 (73.8%) underwent SLNB only and 1529 (26.2%) underwent ALND (± preceded by SLNB). From 2006 to 2016, the rate of SLNB only (not followed by ALND) increased from 11 to 94% (*p* < 0.001) (see Fig. 1). The rate of ALND decreased in both patient groups with ypN0 and ypN+ status (see Table 2). In the patients in whom SLNB was not followed by ALND, the proportion of patients with positive SLNs increased from 7 to 19%.

**Table 2:** Overview of decrease in ALND rates for subgroups based on ypN status for both cN0 and cN + patients (all patients of the cohort were included in this analysis)

Year of diagnosis	2006	2007	2008	2009	2010	2011	2012	2013	2014	2015	2016
cN0 patients, *n*	164	138	191	185	303	523	610	831	943	1280	662
ALND rate in:											
ypN0, % (absolute numbers)	78.3 (65/83)	69.7 (62/89)	63.2 (67/106)	44.2 (42/95)	31.9 (59/185)	13.1 (37/282)	6.1 (22/359)	3.8 (20/520)	3 (19/626)	2.4 (22/927)	1.2 (6/512)
ypN+ , % (absolute numbers)	100 (81/81)	95.9 (47/49)	91.8 (78/85)	92.2 (83/90)	88.1 (104/118)	75.9 (183/241)	61.8 (155/251)	50.5 (157/311)	31.2 (99/317)	24.6 (87/353)	22.7 (34/150)

Year of diagnosis	2006	2007	2008	2009	2010	2011	2012	2013	2014	2015	2016
cN + patients, *n*	302	363	456	487	487	606	608	744	806	1120	652
ALND rate in:											
ypN0, % (absolute numbers)	98.2 (55/56)	98.8 (85/86)	100 (103/103)	100 (82/82)	93.6 (102/109	92.1 (116/126	94 (125/133)	80 (133/166)	65.9 (116/176)	45.2 (109/241)	41.9 (49/117)
ypN+ , % (absolute numbers)	99.6 (245/246)	99.6 (276/277)	100 (353/353)	99.8 (404/405)	100 (378/378)	97.9 (470/480)	97.3 (462/475)	93.6 (541/578)	84.9 (535/630)	61.7 (542/879)	55.5 (297/535)

SLNB was performed prior to compared with after NST in 3401 (65%) and 1815 (35%) cases, respectively. Over time, SLNB was increasingly performed after NST (33% in 2006 vs. 62% in 2016, *p* < 0.001). The overall rate of completion ALND was 23.4% when SLNB was performed prior to NST and 6.7% when SLNB was performed after NST (*p* < 0.001). For SLNB performed prior to NST, the rate of completion ALND decreased from 58 to 12%; for SLNB performed after NST the rate of completion ALND decreased from 46 to 2% (see Fig. 1).

Overall, 4288 of 5830 cN0 patients (74%) were treated with adjuvant radiotherapy (80% in 2006 and 68% in 2016). Data on radiotherapy fields were unknown for the majority of cN0 patients until 2010. From 2011 until 2016, data on radiotherapy fields were known for 91.7% (3244/3539) of cN0 patients treated with adjuvant radiotherapy, ranging from 84 to 95.8% dependent on the year of diagnosis. Adjuvant radiotherapy included *regional* radiotherapy in 36% (122/339) of cN0 patients treated with radiotherapy in 2011 and in 26% (100/381) of cN0 patients treated with radiotherapy in 2016. Out of the 3244 cN0 patients treated from 2011 until 2016 with known radiotherapy fields, 72% of patients with an ypN+ status received regional radiotherapy compared to 6% of patients with an ypN0 status (*p* < 0.001).

### cN+ patients

**Fig. 2:**
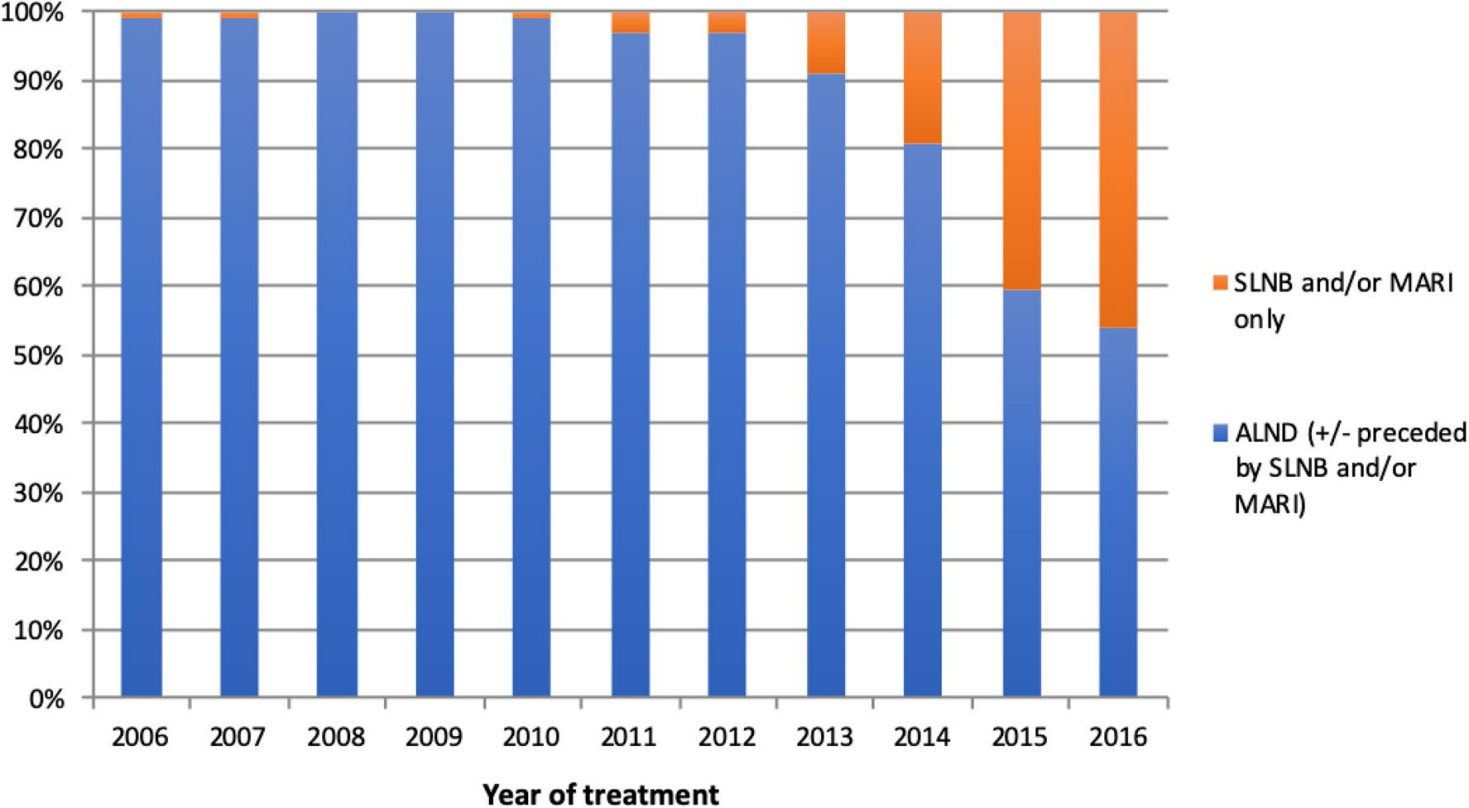
Course over time for axillary staging in cN+ patients treated with NST

Nodal status was positive at the time of diagnosis (cN+) in 6631 patients (53.2%). In total, 1054 (16%) underwent SLNB and/or MARI only and 5577 cN+ patients (84%) underwent ALND (± preceded by SLNB and/or MARI). From 2006 to 2016, the rate of SLNB and/or MARI only increased from 1 to 46% and the rate of (completion) ALND decreased from 99 to 54% (*p* < 0.001) (see Fig. 2). Over this period, the rate of ALND decreased from 98% (55/56) to 42% (48/115) in cN+ ypN0 patients and from 100 to 56% (296/526) in cN+ ypN+ patients (see Table 2). In 2016, 372 cN+ patients (58%) underwent staging by SLNB and/or MARI: in 294 patients (79%), this was not followed by ALND.

In multivariable logistic regression analysis, the following variables were associated with significantly decreased odds ratios for ALND: year of diagnosis (OR 0.47, CI 0.44–0.50) and HER2-positive disease (OR 0.62, CI 0.52–0.75). The following variables were associated with significantly increased odds ratios for ALND: age (OR 1.01, CI 1.00–1.02), clinical tumor stage (T2 vs. T1 OR 1.32, CI 1.06–1.65, T3 vs. T1 OR 2.04, CI 1.58–2.63, T4 vs. T1 OR 6.37, CI 4.26–9.50), and clinical nodal stage (N3 vs. N1 OR 1.65, CI 1.28–2.12) (see Table 3).

**Table 3:** Univariable and multivariable analysis for performing ALND in cT1-4N+ patients treated with NST

	Number of cases with ALND (%)	Univariable analysis		Multivariable analysis (*n* = 6442)	
		OR (95% CI)	*p* value	OR (95% CI)	*p* value
Year of diagnosis	300/302 (99.3%) in 2006 to 346/652 (53.1%) in 2016	0.48 (0.45–0.50)	*p* < 0.001	0.47 (0.45–0.50)	*p* < 0.001
Age (per year)		1.01 (0.99–1.01)	*p* = 0.051	1.01 (1.00–1.02)	*p* = 0.037
Clinical tumor status					
T1	635/845 (75.1%)	Reference		Reference	
T2	2411/2996 (80.5%)	1.36 (1.14–1.63)	*p* = 0.001	1.32 (1.06–1.63)	*p* = 0.013
T3	1391/1611 (86.3%)	2.09 (1.69–2.58)	*p* < 0.001	2.00 (1.56–2.58)	*p* < 0.001
T4	1127/1166 (96.7%)	9.56 (6.70–13.63)	*p* < 0.001	6.49 (4.36–9.66)	*p* < 0.001
Clinical node status					
N1	4697/5582 (84.1%)	Reference		Reference	
N2	220/257 (85.6%)	1.12 (0.79–1.60)	*p* = 0.531	1.31 (0.87–1.96)	*p* = 0.197
N3	658/759 (86.7%)	1.23 (0.93–1.53)	*p* = 0.070	1.66 (1.29–2.14)	*p* < 0.001
ER positive		0.97 (0.84–1.12)	*p* = 0.703		
No	1831/2157 (84.9%)				
Yes	3599/4258 (84.5%)				
HER2 positive		0.83 (0.72–0.96)	*p* = 0.013	0.62 (0.52–0.73)	*p* < 0.001
No	3920/4604 (85.1%)				
Yes	1532/1851 (82.7%)				

*OR* odds ratio, *CI* confidence interval

Overall, 5.824 out of 6631 cN+ patients (88%) were treated with adjuvant radiotherapy. Data on radiotherapy fields were unknown for the majority of cN+ patients until 2010. From 2011 until 2016, data on radiotherapy fields were known for 91.9% (3671/3996) of cN+ patients treated with adjuvant radiotherapy, ranging from 83.6% to 97% dependent on the year of diagnosis. Adjuvant radiotherapy included *regional* radiotherapy in 67.8% (318/469) of cN+ patients treated with radiotherapy in 2011 and in 80% (368/458) of cN+ patients treated with radiotherapy in 2016. Out of the 3671 cN+ patients treated from 2011 until 2016 with known radiotherapy fields, 2251/2955 (76%) of patients with an ypN+ status received regional radiotherapy compared to 424/716 (59%) of patients with an ypN0 status (*p* < 0.001). In 2955 cN +  ypN + patients, 73% received regional radiotherapy when ALND was performed compared to 85% when ALND was not performed. In 716 cN +  ypN0 patients, 57% received regional radiotherapy when ALND was performed compared to 62.5% when ALND was not performed).

### Trends by hospital type

Overall, 823 (6.6%), 6457 (51.8%), and 5181 (41.6%) patients were treated in academic hospitals, teaching hospitals, and community hospitals, respectively. From 2006 to 2016, an overall decrease in ALND was observed from 97 to 38% in academic hospitals (*p* < 0.001), from 94 to 27% in teaching hospitals (*p* < 0.001) and from 97 to 32% in community hospitals (*p* < 0.001). In 2016, staging was performed by means of SLNB and/or MARI in 53% (16/30) of cN+ patients in academic hospitals, 61% (216/354) in teaching hospitals, and 52% (140/268) in community hospitals (*p* = 0.083). In these patients, SLNB and/or MARI was not followed by ALND in 75% (12/16), 75% (161/216), and 86% (121/140), respectively (*p* = 0.025). In 2016, the overall axillary pCR rate in cN+ patients was 17.9%.

## Discussion

This large Dutch population-based cohort study showed that axillary surgery has changed considerably in daily practice over the past decade in cT1-4N0-3 breast cancer patients treated with NST. In cN0 patients, a substantial decrease in ALND and increase in SLNB only was observed, with SLNB being increasingly performed after NST. Additionally, this study revealed that ALND has increasingly been omitted after NST in cN+ patients.

In patients who undergo adjuvant systemic therapy, indications for omitting ALND in cN0 patients have extended over the years from patients with negative SLNS(s) to patients with positive SLN(s) [15]. Even 10-year survival outcomes from the ACOSOG Z0011 and IBCSG 23-01 trials corroborated non-inferiority of SLNB alone compared to ALND for patients with limited positive SLN(s) [4, 16]. Abandoning completion ALND in cN0 patients with positive SLN(s) treated with adjuvant systemic therapy is already ongoing for years, even prior to the publication of the abovementioned trials [17–20]. Ever since the publication of these trial results, implementation of SLNB only in this population is expanding [11, 21]. The current study shows that abandoning completion ALND in cN0 patients with positive SLN(s) is also taking place in patients treated with NST, even though results of the previous trials only apply to patients treated with *adjuvant* systemic therapy. Over the past decade, SLNB was increasingly performed after NST rather than prior to NST. Several previous studies demonstrated that performing SLNB after NST is associated with lower rates of a positive SLNB [22–24]. Thus, ALND can be omitted more often when SLNB is performed after NST. Hence, the change in timing of SLNB found in this study contributed to the decreasing use of ALND.

The results presented here prove that clinicians are willing to adopt a SLNB-only strategy in the neoadjuvant setting as well, even though such a strategy is not evidence-based. Whether SLNB alone instead of the routine use of ALND in patients with limited positive SLN(s) treated in the *neoadjuvant* rather than adjuvant setting provides similar results in terms of overall survival is yet unknown. One should bear in mind that in contrast to the adjuvant setting, positive SLN(s) in the neoadjuvant setting indicates therapy-resistant disease and may represent a different tumor biology. Since long-term follow-up of cN0 patients with positive SLNs that did not undergo completion ALND in the neoadjuvant setting is lacking, it is too early to tell whether it is safe to consider and treat these groups similarly. Furthermore, it is unknown to what extent ALND has been replaced by regional radiotherapy in these patients.

Regarding cN+ patients treated with NST, several less invasive procedures have been proposed over the past years in an attempt to prevent unnecessary ALND in patients who achieve axillary pCR: SLNB, excision of a pre-treatment marked positive lymph node (like MARI) or a combination of these two procedures (like Targeted Axillary Dissection and RISAS) [13, 25–27]. Despite limited evidence for the safety of replacing ALND by these procedures, implementation of such strategies is occurring worldwide [28]. The results of the current study confirm this trend: from 2013 (prior to publications on accuracy of MARI and TAD), rates of SLNB and/or MARI for axillary staging after NST started increasing up to 58% in 2016. The increase in rates of SLNB and/or MARI in cN+ patients was present in all three types of hospitals. Although SLNB and/or MARI is offered more and more to cN+ patients in order to omit ALND in case of axillary pCR, 42% of patients with an axillary pCR still underwent (completion) ALND in 2016. This indicates that selecting the right patient for the appropriate procedure is challenging. At the same time, completion ALND may have been performed as part of validating studies. In some institutions, ALND is performed in all cN+ patients, irrespective of response to NST, which may also in part explain this finding. Again, it is unknown to what extent ALND has been replaced by regional radiotherapy.

Notably, the decrease in ALND rates in cN+ patients treated with NST is also present in patients who do not achieve an axillary pCR. In 2016, 44% of the cN+ patients with post-NST residual axillary disease did not undergo completion ALND. Multiple studies reported improved survival for patients with a complete response (ypT0/ypN0) to NST compared to patients without a complete response [29, 30]. These results indicate the necessity of treatment escalation rather than de-escalation in patients without axillary pCR to improve prognosis, especially in certain subtypes (such as triple negative breast cancer) [31]. Whether current practices of omitting ALND in cN+ patients without axillary pCR will negatively impact prognosis is yet unknown. In the Alliance 11,202 trial, prognosis of cN + patients with positive SLN(s) after NST is compared between those treated with completion ALND and those treated with axillary radiotherapy [32].

Although the current study describes a large cohort of patients, several limitations have to be taken into account. Regarding adjuvant treatment plans, sufficient data on radiotherapy fields were not available for the whole cohort. It is expected that simultaneously with the decreasing rates of ALND, rates of regional radiotherapy increase. This study did suggest such a trend in cN+ patients, but further research is needed for a thorough assessment of (regional) radiotherapy administration in patients treated with NST. Furthermore, the impact of omitting ALND on prognosis in terms of overall and disease-free survival could not be assessed since data on recurrences are not (yet) available for this cohort.

To conclude, axillary surgical staging changed significantly with a major decrease in ALND rates in breast cancer patients treated with NST over the past decade. However, selecting the right patients for whom omitting ALND is oncologic safe appears challenging, especially in pre-treatment cN+ patients and patients with residual axillary disease. Studies assessing long-term prognosis of such patients in whom ALND is omitted are urgently needed.

## References

[1] Mougalian SS, Soulos PR, Killelea BK (2015). Use of neoadjuvant chemotherapy for patients with stage I to III breast cancer in the United States. Cancer.

[2] Vugts G, Maaskant-Braat AJ, Nieuwenhuijzen GA, Roumen RM, Luiten EJ, Voogd AC (2016). Patterns of care in the administration of neo-adjuvant chemotherapy for breast cancer. A population-based study. Breast J.

[3] Giuliano AE, Ballman K, McCall L (2016). Locoregional recurrence after sentinel lymph node dissection with or without axillary dissection in patients with sentinel lymph node metastases: long-term follow-up from the American College of Surgeons Oncology Group (Alliance) ACOSOG Z0011 randomized trial. Ann Surg.

[4] Giuliano AE, Ballman KV, McCall L (2017). Effect of axillary dissection vs no axillary dissection on 10-year overall survival among women with invasive breast cancer and sentinel node metastasis: the ACOSOG Z0011 (Alliance) Randomized Clinical Trial. JAMA.

[5] Giuliano AE, Hunt KK, Ballman KV (2011). Axillary dissection vs no axillary dissection in women with invasive breast cancer and sentinel node metastasis: a randomized clinical trial. JAMA.

[6] Giuliano AE, McCall L, Beitsch P (2010). Locoregional recurrence after sentinel lymph node dissection with or without axillary dissection in patients with sentinel lymph node metastases: the American College of Surgeons Oncology Group Z0011 randomized trial. Ann Surg..

[7] Galimberti V, Cole BF, Zurrida S (2013). Axillary dissection versus no axillary dissection in patients with sentinel-node micrometastases (IBCSG 23–01): a phase 3 randomised controlled trial. Lancet Oncol.

[8] Donker M, van Tienhoven G, Straver ME (2014). Radiotherapy or surgery of the axilla after a positive sentinel node in breast cancer (EORTC 10981–22023 AMAROS): a randomised, multicentre, open-label, phase 3 non-inferiority trial. Lancet Oncol.

[9] Dengel LT, Van Zee KJ, King TA (2014). Axillary dissection can be avoided in the majority of clinically node-negative patients undergoing breast-conserving therapy. Ann Surg Oncol.

[10] Mamtani A, Patil S, Van Zee KJ (2016). Age and receptor status do not indicate the need for axillary dissection in patients with sentinel lymph node metastases. Ann Surg Oncol.

[11] Poodt IGM, Spronk PER, Vugts G (2018). Trends on axillary surgery in nondistant metastatic breast cancer patients treated between 2011 and 2015: a Dutch population-based study in the ACOSOG-Z0011 and AMAROS era. Ann Surg.

[12] Tsao MW, Cornacchi SD, Hodgson N (2016). A population-based study of the effects of a regional guideline for completion axillary lymph node dissection on axillary surgery in patients with breast cancer. Ann Surg Oncol.

[13] van Nijnatten TJ, Schipper RJ, Lobbes MB, Nelemans PJ, Beets-Tan RG, Smidt ML (2015). The diagnostic performance of sentinel lymph node biopsy in pathologically confirmed node positive breast cancer patients after neoadjuvant systemic therapy: a systematic review and meta-analysis. Eur J Surg Oncol.

[14] Schouten LJ, Höppener P, van den Brandt PA, Knottnerus JA, Jager JJ (1993). Completeness of cancer registration in Limburg, The Netherlands. Int J Epidemiol.

[15] Francissen CM, Dings PJ, van Dalen T, Strobbe LJ, van Laarhoven HW, de Wilt JH (2012). Axillary recurrence after a tumor-positive sentinel lymph node biopsy without axillary treatment: a review of the literature. Ann Surg Oncol.

[16] Galimberti V, Cole BF, Viale G (2018). Axillary dissection versus no axillary dissection in patients with breast cancer and sentinel-node micrometastases (IBCSG 23–01): 10-year follow-up of a randomised, controlled phase 3 trial. Lancet Oncol.

[17] Bilimoria KY, Bentrem DJ, Hansen NM (2009). Comparison of sentinel lymph node biopsy alone and completion axillary lymph node dissection for node-positive breast cancer. J Clin Oncol.

[18] Boughey JC (2014). Axillary dissection can be avoided in the majority of clinically node-negative patients undergoing breast-conserving therapy, by Dengel et al. Ann Surg Oncol.

[19] Yi M, Giordano SH, Meric-Bernstam F (2010). Trends in and outcomes from sentinel lymph node biopsy (SLNB) alone vs SLNB with axillary lymph node dissection for node-positive breast cancer patients: experience from the SEER database. Ann Surg Oncol.

[20] Hieken TJ, Boughey JC (2013). Axillary dissection versus no axillary dissection in patients with sentinel-node micrometastases: commentary on the IBCSG 23–01 Trial. Gland Surg.

[21] Weiss A, Mittendorf EA, DeSnyder SM (2018). Expanding implementation of ACOSOG Z0011 in surgeon practice. Clin Breast Cancer.

[22] Hunt KK, Yi M, Mittendorf EA (2009). Sentinel lymph node surgery after neoadjuvant chemotherapy is accurate and reduces the need for axillary dissection in breast cancer patients. Ann Surg.

[23] Fontein DB, van de Water W, Mieog JS, Liefers GJ, van de Velde CJ (2013). Timing of the sentinel lymph node biopsy in breast cancer patients receiving neoadjuvant therapy—recommendations for clinical guidance. Eur J Surg Oncol.

[24] van Diest PJ, de Munck L, Sonke GS (2015). Population based study on sentinel node biopsy before or after neoadjuvant chemotherapy in clinically node negative breast cancer patients: Identification rate and influence on axillary treatment. Eur J Cancer.

[25] Caudle AS, Yang WT, Krishnamurthy S (2016). Improved axillary evaluation following neoadjuvant therapy for patients with node-positive breast cancer using selective evaluation of clipped nodes: implementation of targeted axillary dissection. J Clin Oncol.

[26] Donker M, Straver ME, Wesseling J (2015). Marking axillary lymph nodes with radioactive iodine seeds for axillary staging after neoadjuvant systemic treatment in breast cancer patients: the MARI procedure. Ann Surg.

[27] van Nijnatten TJA, Simons JM, Smidt ML (2017). A novel less-invasive approach for axillary staging after neoadjuvant chemotherapy in patients with axillary node-positive breast cancer by combining radioactive iodine seed localization in the axilla with the sentinel node procedure (RISAS): a Dutch prospective multicenter validation study. Clin Breast Cancer.

[28] Diego EJ, McAuliffe PF, Soran A (2016). Axillary staging after neoadjuvant chemotherapy for breast cancer: a pilot study combining sentinel lymph node biopsy with radioactive seed localization of pre-treatment positive axillary lymph nodes. Ann Surg Oncol.

[29] Cortazar P, Zhang L, Untch M (2014). Pathological complete response and long-term clinical benefit in breast cancer: the CTNeoBC pooled analysis. Lancet.

[30] Mougalian SS, Hernandez M, Lei X (2016). Ten-year outcomes of patients with breast cancer with cytologically confirmed axillary lymph node metastases and pathologic complete response after primary systemic chemotherapy. JAMA Oncol.

[31] Yang TJ, Morrow M, Modi S (2015). The effect of molecular subtype and residual disease on locoregional recurrence in breast cancer patients treated with neoadjuvant chemotherapy and postmastectomy radiation. Ann Surg Oncol.

[32] NCT01901094 (2018) Comparison of axillary lymph node dissection with axillary radiation for patients with node-positive breast cancer treated with chemotherapy. principal investigator: Judy Boughey. Mayo Clinic. https://clinicaltrials.gov/ct2/show/NCT01901094. Accessed 5 Nov 2018

